# Age- and Sex-Dependent Effects of Early Life Stress on Hippocampal Neurogenesis

**DOI:** 10.3389/fendo.2014.00013

**Published:** 2014-02-20

**Authors:** Manila Loi, Sylwia Koricka, Paul J. Lucassen, Marian Joëls

**Affiliations:** ^1^Department of Translational Neuroscience, Brain Center Rudolf Magnus, University Medical Center Utrecht, Utrecht, Netherlands; ^2^Swammerdam Institute for Life Sciences – Center for Neuroscience, University of Amsterdam, Amsterdam, Netherlands

**Keywords:** maternal deprivation, maternal separation, stress, rat, dentate gyrus, adult neurogenesis, proliferation, hippocampus

## Abstract

Early life stress is a well-documented risk factor for the development of psychopathology in genetically predisposed individuals. As it is hard to study how early life stress impacts human brain structure and function, various animal models have been developed to address this issue. The models discussed here reveal that perinatal stress in rodents exerts lasting effects on the stress system as well as on the structure and function of the brain. One of the structural parameters strongly affected by perinatal stress is adult hippocampal neurogenesis. Based on compiled literature data, we report that postnatal stress slightly enhances neurogenesis until the onset of puberty in male rats; when animals reach adulthood, neurogenesis is reduced as a consequence of perinatal stress. By contrast, female rats show a prominent reduction in neurogenesis prior to the onset of puberty, but this effect subsides when animals reach young adulthood. We further present preliminary data that transient treatment with a glucocorticoid receptor antagonist can normalize cell proliferation in maternally deprived female rats, while the compound had no effect in non-deprived rats. Taken together, the data show that neurogenesis is affected by early life stress in an age- and sex-dependent manner and that normalization may be possible during critical stages of brain development.

## Early Life Stress and Brain Development

Early life represents a critical phase in brain development as many regions are not fully formed at birth or undergo extended postnatal maturation. The dentate gyrus (DG), part of the hippocampal formation, is an extreme case where the majority of neurons are generated after birth (1). The continued formation of new neurons after birth, known as adult neurogenesis, is restricted to a limited number of brain areas: in addition to the DG, neurogenesis occurs in the subventricular zone (SVZ) and in the olfactory bulb (2). Even in other parts of the brain, growth is not completed at birth. For instance, the prefrontal cortex (PFC) continues to develop well into adulthood (3). Cortical thickness in humans reaches a maximum around age 35 (4). In addition to the progressive growth until adulthood, new connections continue to be formed, too. The intricate formation and pruning of essential contacts eventually lead to an effective connectome and functional network (5).

**Figure 1:**
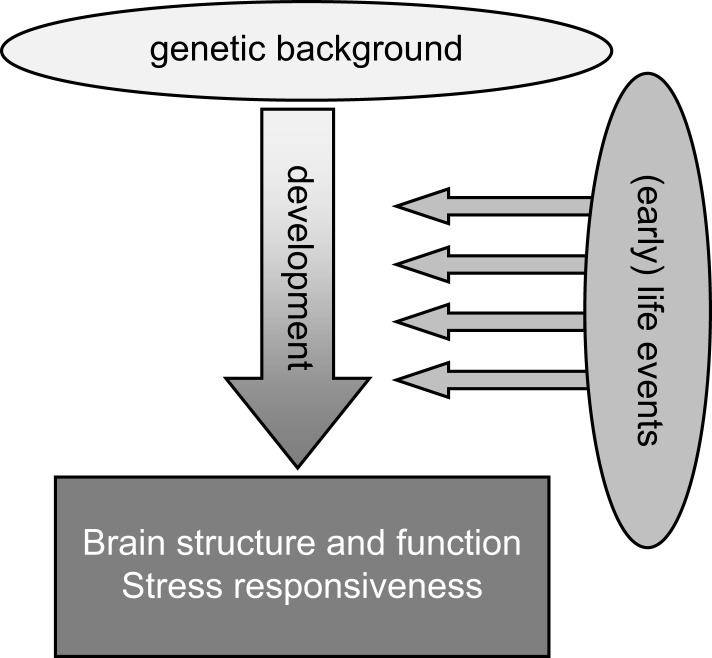
**General scheme highlighting that life events, especially when experienced during the early developmental stage, may strongly impact the development of the brain, especially in genetically susceptible individuals**. These gene–environment interactions during development will strongly contribute to the overall brain structure and function as well as stress responsiveness in adulthood.

It is therefore not surprising that potential or actual perturbations in the individual’s environment and “homeostasis” – subjectively experienced as “stress” – particularly when these take place during the critical phase of early development, can have important lasting consequences for brain structure and function later in life (Figure 1). In interaction with the genetic profile, early environmental influences “shape” brain maturation as well as the way in which an individual deals with environmental challenges throughout the rest of life. In humans, brain structure and function as well as the ability to cope with stress together determine the vulnerability to psychopathology. Retrospective case–control studies for various psychiatric illnesses, including post-traumatic stress disorder (PTSD) (6), depression (7), schizophrenia (8), and also borderline syndrome (9), have consistently shown that early life adversity is a significant risk factor. The risk increases when early life adversity is severe, prolonged, repeated, and/or characterized by a lack of control over the situation (10). Prospective studies, though more rare, confirm this view [see e.g., Ref. (11–13)].

The sequential steps through which early life adversity changes brain structure and function in a lasting manner, and hence the later risk for psychopathology, is hard to investigate in human subjects, given the long duration of brain maturation, the restrictions in obtaining detailed information about signal transduction in the brain and the lack of control over both genetic and environmental factors. Therefore, research has resorted to animal models, which have fewer of these drawbacks.

In rodents, many models for early life adversity have been developed (14, 15). Some intervene with the prenatal environment, e.g., by stressing the pregnant female (16–19) or by exposing her to compounds acting on stress hormone receptors, e.g., dexamethasone (20). The majority of models, though, focus on the postnatal environment. Since the care provided by the dam represents a strong environmental influence during the first postnatal weeks, many models have specifically concentrated on (disturbed) mother–pup interactions. One model, developed largely by Michael Meaney and coworkers, makes use of natural variations in maternal care provided by the dams (21). Their licking and grooming behavior shows a classic normal distribution, with the majority of the mothers providing moderate amounts of care (22). However, some mothers show extremely high or low amounts of licking–grooming behavior (>1 standard deviation above or below the mean respectively). Their offspring can be examined in adulthood and even into the next generation to study consequences of maternal care (22). Notably, through cross-fostering, the influence of maternal care can be dissociated from the contributions of the genetic background (23).

Many other models actively intervene with the mother–pup interaction, e.g., by limiting the bedding and nesting material in the cage, which induces fragmented care in the dam (24). This, in turn, has lasting consequences for brain development and behavior in the offspring. Separation of the pups from the mother has also been applied in various models. This can range from brief, daily separations to more extreme conditions where the dam and her offspring are separated for up to 24 h. Brief separations, e.g., 15 min handling of the pups during the first postnatal weeks, actually results in an overall enhancement of maternal care, because the mother bestows more care on her pups upon their return to the cage; this has been used as a model for environmental enrichment (25). More prolonged separations, e.g., for 3 h daily between postnatal days (PND) 2 and 14 or deprivation of the mother for 24 h at PND 3 or 9, can be considered models for impoverished and poor care or even neglect (26).

Studies using these models have shown that many aspects of brain structure and function are strongly affected by early life adversity, some of which can be normalized by environmental enrichment (27, 28). One set of data pertains to the development of the stress response itself. Stress rapidly activates the autonomic nervous system, eventually causing the release of (nor)adrenaline from the adrenal medulla. Slightly later, the hypothalamic–pituitary–adrenal (HPA) axis is activated, which leads to release of corticosteroid hormones (corticosterone in rodents and cortisol in humans) from the adrenal cortex into the circulation; this response is terminated 1–2 h later by negative feedback actions of corticosteroids at the level of the pituitary gland, the hypothalamus, and extra-hypothalamic regions (29, 30). Thus, after stress, the brain is exposed to successive waves of noradrenaline and (slightly later) corticosteroid hormones, which have their own and combined roles in mediating effects of stress on the brain, that normalize some hours later (31). In the brain, corticosteroids bind to discretely localized intracellular receptors, most notably the glucocorticoid receptor (GR) that is enriched in hippocampal CA1 and dentate granule cells (29). Corticosteroids also bind to another receptor, the mineralocorticoid receptor, but the affinity for this receptor is very high, so that it is substantially bound to corticosteroids already under conditions of rest (29, 32). Early life adversity was found to reduce the number of hippocampal GRs and impair the negative feedback, causing prolonged exposure of the brain to corticosteroids in the aftermath of stress (33).

In addition to effects on stress responsiveness, also brain structure and function are affected by the early life environment (26). Many studies have shown that, e.g., the complexity of dendritic trees, the number of synaptic contacts and growth factor levels depend on early life history, although the direction of these effects can be region dependent (34). Similarly, the extent of neurogenesis in adolescence and adulthood is affected by circumstances experienced earlier in life (7). In this paper, we will highlight the effects of the early life environment on hippocampal neurogenesis (see below), focusing on stress experienced just prior to, or during, the first 2 postnatal weeks.

Not only structural plasticity but also functional plasticity has been the subject of study. For instance, hippocampal long-term potentiation, i.e., the prolonged strengthening of synaptic contacts, which is thought to underlie memory formation (35), is generally impaired in adult rats that experienced fragmented or low levels of maternal care, or were separated from the mother during the first postnatal weeks (36, 37). These structural and functional changes contribute to behavioral changes. For instance, contextual hippocampus-dependent memory is impaired in adult rats that experienced reduced amounts of maternal care, be it due to natural variations (27, 37, 38) or imposed by separation (39, 40). However, other cognitive domains – e.g., decision making or reward processes that depend on an optimal function of the PFC – are also disturbed in adult rodents with a history of early life adversity (41, 42).

The overall adaptive value of these long-term changes in stress responsiveness, brain structure and function after early life adversity can be best appreciated when studying individuals under various circumstances later in life. For instance, long-term potentiation and hippocampus-dependent learning are impaired under non-stressful experimental conditions in adult offspring from low licking–grooming mothers, or in animals with a history of 24 h of MD at PND 3 (37, 38, 40, 43). In contrast, when these animals are tested under conditions of elevated corticosterone levels, long-term potentiation and hippocampus-dependent learning are actually improved (37, 38, 40). This suggests that the early life environment may affect brain development in such a way that the network can optimally perform under comparable, i.e., matching, conditions later in life. Inadequate responses may arise when there is a mismatch between early life, and the predictions, or “settings” based on that environment on the one hand, and the actual, later life conditions the individual experiences at an adult age on the other hand (44–46).

## Neurogenesis

Adult neurogenesis refers to the formation of new, functional neurons that originate from stem cells present in the adult brain. This form of structural plasticity occurs in at least two brain regions; the SVZ of the lateral ventricles, from where cells migrate through the rostro-migratory stream toward the olfactory bulb, and in the subgranular zone (SGZ) of the hippocampal DG. Adult neurogenesis is strongly affected by the early life environment (7, 47, 48). Whereas in the SVZ, the newborn cells participate in olfactory learning, and newborn cells in the DG have been implicated in specific aspects of spatial memory formation and cognition such as pattern separation (49).

During the dynamic process of neurogenesis, stem cells go through several, distinct stages of development (50). Following an initial phase of proliferation during which the initial stem cell pool mainly undergoes expansion, a selection process occurs after approximately 1 week during which around 50% of the newly generated cells die through apoptosis. The surviving cells use radial glia cells as a scaffold to migrate into the granule cell layer, where they eventually differentiate into a mature neuronal phenotype. The proliferation phase is often studied using immunocytochemical markers like Ki-67 or proliferating cell nuclear antigen, while the differentiation phase is usually investigated with doublecortin (DCX), a microtubule-associated protein expressed in young migratory neurons (51). The spatio-temporal expression pattern of DCX largely coincides with the process of adult neurogenesis in the rat hippocampus. Cell survival and cell fate can be studied several weeks after (intra-peritoneal) pulse labeling with bromo-deoxy-uridine (BrdU), a compound that is incorporated into the DNA of dividing cells. The fate and progeny of BrdU-incorporating cells can then later be monitored using double-immunofluorescent labeling with markers for mature neuronal or glial cells. With viral vectors, which label only dividing cells, it has been shown that most adult-born cells, 3–4 weeks after their birth, express adult neuronal markers and are functionally incorporated within the existing DG network (52).

The process of neurogenesis is regulated by several environmental factors including enriched environmental housing or physical exercise, both stimulating the survival of the newborn neurons (53). By contrast, aging and exposure to acute or chronic stress strongly suppress one or more phases of the neurogenic process (54).

Elevated stress hormone levels or an activated HPA-axis is commonly observed in depressed patients. Recent studies have further suggested that also impairments in structural plasticity, including neurogenesis, may be involved in the pathophysiology of depression and in the hippocampal volume reduction in this disorder (55). This “neurogenic theory” of depression postulates that a suppressed rate of cyto- or neurogenesis contributes to the (vulnerability for) depression (56–58), and is supported by the findings that: (1) stress inhibits neurogenesis in animals and is a risk factor for depression; (2) depressed patients often display hippocampal volume reductions parallel to cognitive deficits and HPA activation; (3) most antidepressant drugs do not exert their therapeutic effect until after 3–4 weeks of administration, a time-to-effect that parallels the maturation period of adult newborn neurons; (4) many antidepressants increase or normalize reductions in neurogenesis, particularly in young animals; and (5) disruption of neurogenesis blocks the behavioral response to antidepressant drugs (59, 60). However, this theory is not always supported and still under debate (58, 61, 62).

While stress-induced reductions of neurogenesis occurring during adulthood are generally reversible, e.g., after appropriate recovery periods or antidepressant drug treatment, the changes induced by *early life* stress are generally longer lasting and the consequences often persist throughout life (see further below). One reason for this difference could be that stress occurring during early life interferes with the development of the DG, which is largely postnatal in rodents. However, it remains poorly understood why such deficits are so long-lasting and whether they can be prevented or reversed at all.

## Age- and Sex-Dependent Effects of Perinatal Stress

Adult neurogenesis is sensitive to the early life environment. As summarized in Table 1, perinatal stress in male rats was generally found to suppress neurogenesis (17, 63). The effects appear to be *region-specific*: for instance, prenatal stress impaired neurogenesis in the DG but not in the olfactory bulb (64).

**Table 1:** **Overview of the effects of perinatal stress on cell proliferation and neurogenesis in rodents, as measured at various times in life and as a function of sex**.

Type of early life intervention	Sex	Age during stress	Age when neurogenesis is studied	Marker	Effect on neurogenesis	Reference
Prenatal stress: restraint stress	Males	Gestational day: 14–21	PND 28	BrdU, used as cell proliferation marker	Reduction	Koehl et al. (65)
			3 months	
			10 months	
			22 months	

Prenatal stress: restraint stress	Males	Gestational day: 15–delivery	4 months	DCX, Ki-67, BrdU injected 1 day before the animals were sacrificed	Reduction	Lemaire et al. (18)
		Gestational day: 15–delivery	6 months	BrdU injected 3 weeks before the animals were sacrificed	
	Males	Gestational day: 15–delivery	26 months	DCX, Ki-67, BrdU injected 1 day before the animals were sacrificed	

Prenatal stress: restraint stress or randomized stressors	Males vs. Females	Gestational day: 14–21	5–6 months	DCX	Reduction in females (controls and stressed group)	Mandyam et al. (66)
	Males	Gestational day: 14–21	5–6 months	Ki-67	Reduction	
	Females	Gestational day: 14–21	5–6 months	Ki-67	Reduction	

Prenatal stress: interaction with resident + restraint stress	Males (from selective line LAB and HAB)	Gestational day: 5–20	PND 43	DCX	Reduction in HAB	Lucassen et al. (63)
					No effect in LAB	
				BrdU injected at PND 11	Reduction in HAB	
					No effect in LAB	

Prenatal stress: restraint stress (animals were tested in behavioral task before to assess neurogenesis)	Males	Gestational day: 15–21	PND 42	DCX	Reduction	Rayen et al. (67)
				Ki-67	
	Females	Gestational day: 15–21	PND 42	DCX	
				Ki-67	

Prenatal stress: restraint stress	Males and females (combined together)	Gestational day: 1–10	PND 40	DCX	Reduction	Madhyastha et al. (68)
	Males and females (combined together)	Gestational day: 11-delivery	PND 40	DCX	

Prenatal nicotine treatment + Maternal separation	Males and females	Gestational day:7–21	PND 14	DCX	Reduction	Wang et al. (69)
		Maternal separation: PND 2–21	
Maternal deprivation (MD)	Males	PND 3 (24 h)	PND 4	Ki-67	No effect	Oomen et al. (70)
	Females	PND 3 (24 h)	PND 4	Ki-67	
	Males	PND 3 (24 h)	PND 21	DCX	Increase	
				Ki-67, BrdU, injected at PND 3	No effect	
	Females	PND 3 (24 h)	PND 21	DCX	Reduction	
				Ki-67, BrdU, injected at PND 3	No effect	

Maternal deprivation (MD)	Males	PND 3 (24 h)	10 weeks	DCX, BrdU, injected at PND 51	Reduction in caudal part of DG but not rostral	Oomen et al. (40)
				Ki-67	Reduction	

Maternal deprivation (MD)	Females	PND 3 (24 h)	10 weeks	DCX, Ki-67, BrdU, injected at PND 51	No effect	Oomen et al. (71)

Maternal separation	Males	PND 1–14: Handling + 180 min. MS	PND 60–70:	BrdU injection: 2 h	Reduction vs. AFR and EH	Mirescu et al. (72)
				BrdU injection: 1 week	Reduction vs. AFR and EH	
				BrdU injection: 3 week	No effect	

Maternal separation	Males	PND 2–14: Handling + 180 min MS	11 weeks	BrdU, injected at 8 weeks for 7 days	No effect compared to EH	Kumar et al. (73)
	Females	PND 2–14: Handling + 180 min MS	11 weeks	BrdU, injected at 8 weeks for 7 days	No effect compared to EH	

Maternal separation (animals were tested in behavioral task before assessing neurogenesis)	Males	PND 2–15 (3 h per day)	± PND 80	DCX, BrdU injection ± PND 65	No effect	Hulshof et al. (74)
				Ki-67	Reduction in ventral but not dorsal hippocampus	

Maternal separation	Males	PND 2–14 (3 h per day)	PND 21	DCX, BrdU, injected 2 h before sacrifice	Increase	Suri et al. (75)
			2 months		No effect	
			15 months		Reduction	

Low licking/grooming	Males	Behavior checked first week of life	PND 8	BrdU injected at PND 7	No effect	Bredy et al. (27)
			PND 21		Reduction	
			PND 90	

Social defeat stress	Males	PND 35–41	PND 42	DCX	Reduction	Buwalda et al. (76)
				BrdU, injected 2 h before sacrifice	

The overall effect of stress on neurogenesis also depends on the *developmental stage* during which the organism experiences stress. Thus, *in utero* exposure to stress or to a variety of pharmacological agents almost invariably reduces neurogenesis in adulthood (Table 1). Postnatal exposure to stress yields more variable results, though suppression of neurogenesis prevails here also.

**Figure 2:**
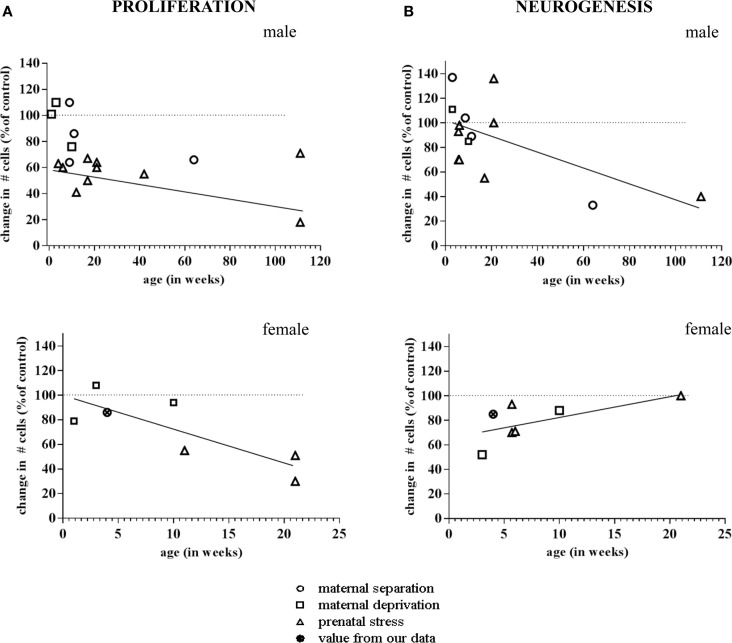
**Meta-analysis of age- and sex-dependent effects of early life adversity on neurogenesis**. The graphs show the percentage change in number of Ki-67 and Doublecortin (DCX)-positive cells after perinatal stress (*y*-axis; 100% is control), as a function of the age at which the change in neurogenesis was determined (*x*-axis, in weeks). **(A)** Both in males (top) and females (bottom), the change in number of Ki-67 positive cells due to prenatal (triangle), maternal separation (diamond) or maternal deprivation (square) was negatively correlated to the age at which the effect was determined. **(B)** In male rodents (top), the number of DCX-positive cells was found to be enhanced by perinatal stress when examined at a very young age. When studied at time-points >2 months of age, generally a decrease in the number of DCX-positive cells was observed. Overall, this resulted in a negative correlation between the effect of early life stress on neurogenesis and the age at which these effects were apparent. In female rats (bottom), we observed a correlation in the opposite direction. The data points are based on the references summarized in Table 1. The one data-point depicted by the filled symbol in the graphs of the females represents the percentage change found in the pilot study described in Figure 4 (not incorporated in Table 1).The striped horizontal line in the graphs indicates the control condition (i.e., the non-stressed groups mentioned in the same publications) against which the number of cells in the stress groups was expressed. The drawn line indicates the best fit for the linear correlation.

**Figure 3:**
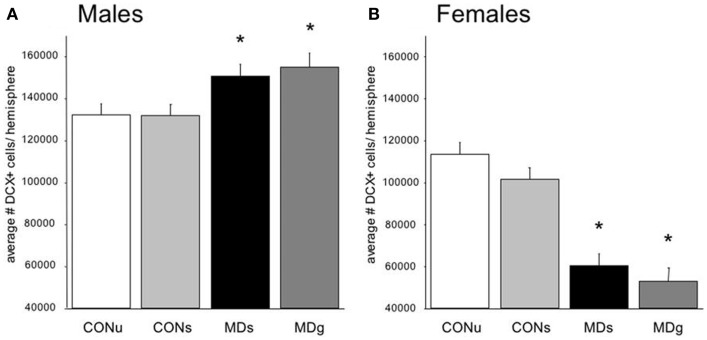
**Sex-dependent effects of maternal deprivation on neurogenesis**. **(A)** The number of Doublecortin (DCX)-positive neurons in the entire dentate gyrus from 21-days-old male rats, which underwent MD for 24 h at postnatal day (PND) 3, was significantly (*p < 0.05; *n* = 7 animals) enhanced compared to the non-deprived controls (CON). Half of the animals received glucose (g) on day 3, to compensate for the loss in nutrients, while the remaining animals received saline (s). There was no effect of sucrose compared to saline treatment. **(B)** The opposite effect was observed in the female littermates: i.e., the number of DCX-positive neurons on PND 21 was significantly reduced in maternally deprived compared to non-deprived rats, regardless of sucrose/saline treatment (*n* = 7). From Ref. (70).

More importantly, the consequences of early life environment depend on the *moment at which neurogenesis is determined*. When tested in adulthood or middle-age, cell proliferation and neurogenesis were usually found to be decreased (Table 1, Figure 2). Yet, at earlier stages, e.g., at PND 21 (70, 75), neurogenesis in males was actually found to be *enhanced* by early life stress (Figure 3), as was BDNF expression and performance in a stressful version of the Morris water maze (75). Apparently, early life adversity can transiently improve dentate functionality, possibly to allow the organism to survive in the adverse conditions. However in the long run, early life adversity seems to program structural plasticity such that it may become a disadvantage, most notably under low to moderately stressful conditions. Overall, this gives rise to a significant negative correlation between the number of proliferating (Ki-67 or BrdU-positive cells; *r*^2^ = −0.464, *p* = 0.05; Pearson test) or DCX-positive (*r*^2^ = −0.623, *p* = 0.017) neurons and age in male rodents.

**Figure 4:**
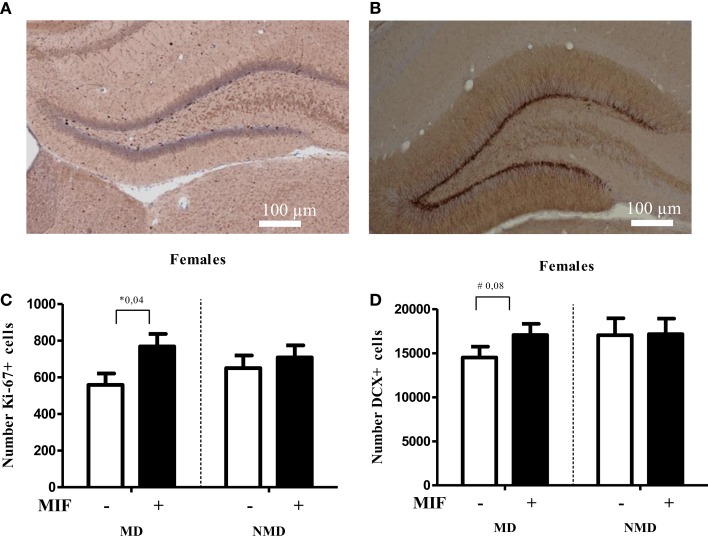
**Brief treatment with the GR antagonist mifepristone protects female rats against the effects of early life stress**. Rats were deprived from their mother for 24 h at PND 3, following the procedure as described in Oomen et al. (70). After weaning at PND 21, they were group-housed with same-sex same-littermates. On PND 26–28 each rat received mifepristone twice daily (5 mg of RU-38486 (Sigma) per 100 g of body weight, dissolved first in 15 μl ethanol and then in 1.5 ml coffee cream (Campina, Woerden, The Netherlands) and administered by oral gavage (77). One day later, at PND 29, female rats were transcardially perfused with saline, followed by 4% paraformaldehyde in phosphate buffer (0.1M; pH 7.4). Tissue handling and staining for DCX and the proliferation marker Ki67 was conducted as described in Oomen et al. (70). **(A)** Typical example of Ki-67 staining in the DG of a control female rat. **(B)** Typical example of DCX staining in the DG of a control female rat. **(C)** Cell proliferation at PND 29, as determined with Ki-67 staining, was significantly increased in the hilus of MD female rats treated with mifepristone (MIF) on PND 26–28, compared to those treated with vehicle. MIF had no effect in non-deprived (NMD) rats. No significant differences between the experimental groups were seen in the dentate as a whole (data not shown). For each animal, we counted the number of Ki67-positive cells in every 10th section and from this the total number of Ki67-positive cells per hemisphere was inferred. All bars represent the mean + SEM per group (*n* = 7–9 animals per group). **(D)** In the supra-pyramidal blade, a trend (*p* = 0.08) toward a significant increase in the number of DCX-positive cells in MD female rats treated with MIF vs. those treated with vehicle was observed. Mifepristone did not alter neurogenesis at all in NMD rats. Though the percentage change in the infrapyramidal blade and the dentate gyrus as a whole showed a comparable pattern, these differences were not significant (*p* > 0.1, data not shown). For each animal, we counted every 10th section sampled in an unbiased stereological manner, yielding up to a total of nine sections per animal. We then expressed the average number of DCX-positive cells per section per animal. All bars represent the mean + SEM per group (*n* = 11–12 animals per group).

Strikingly different effects of early life stress on neurogenesis are seen in female rats (Figure 2). Whereas neurogenesis is enhanced at PND 21 in male rats exposed to 24 h of maternal deprivation at PND 3, a strong suppression was reported in females (Figure 3). However, in females the consequence of early life adversity for the number of DCX-positive cells subsides with age, resulting in an overall positive correlation between the number of DCX-positive cells and age (*r*^2^ = 0.737, *p* = 0.037). By PND 29, the effects of maternal deprivation on neurogenesis are far less prominent than seen at PND 21 [Figure 2 (filled symbol) and Figure 4]. However, the correlation between the change in number of proliferating neurons due to early life adversity and the age at which the effects were determined was comparable between males and females (*r*^2^ = −0.816, *p* = 0.025). This could suggest that in female proliferation of non-neuronal (e.g., glial) cells in adulthood is very sensitive to early life adversity or, *vice versa*, that proliferation of non-neuronal cells is stimulated around weaning, compensating for the loss in young neurons due to perinatal stress.

## Essential Mediators

The molecular pathways through which early life stress can lastingly change stress responsiveness, brain structure and functional performance are only starting to be explored. There is now evidence that epigenetic programing is involved (48, 78), possibly targeting diverse mediators such as NFκB (79), SGK1 (80) or critical steps in the glutamate signaling pathway (81). The GR seems to be a particularly critical element in the cascade leading to lasting changes in brain structure and function. For instance, exon I-7 of the GR promoter is transiently methylated during early development, and again subject to de-methylation after PND 6 (82). This demethylation did not occur in the offspring from low licking and grooming mothers. Temporary treatment with a histone deacetylase inhibitor, a compound that prevents the removal of acetyl groups from histones, thus enabling transcription, could fully prevent the development of the phenotype – characterized by reduced hippocampal GR expression and an impaired negative feedback of the stress induced stress response – in adult offspring of low licking–grooming mothers (82, 83). This suggests that the reduction of GR expression in the offspring of low licking–grooming mothers – and hence corticosteroid over-exposure, particularly after stress – may at least partly be responsible for the structural and functional alterations reported along the lifespan and are likely mediated by epigenetic changes.

If corticosteroid over-exposure is indeed an essential step in the cascade, one would expect to see beneficial effects of treatment with pharmacological agents that block the GR, i.e., the receptor most prominently activated after stress. To test this, we performed a pilot study in which female rats, exposed to maternal deprivation at PND 3, were treated during a critical developmental window with the GR antagonist mifepristone. We selected the period of PND 26–28 for treatment with mifepristone, as earlier studies have shown that interventions at this stage of development have significant consequences for the development of the brain and the response to stress later in life (84). Moreover, we had demonstrated before that even a brief treatment with mifepristone is very powerful in normalizing the effects of chronic stress in adult rats (77).

As shown in Figure 4, the number of Ki67-positive cells was significantly higher in the hilus (but not in the DG as a whole, data not shown) in MD rats treated with mifepristone compared to those treated with vehicle, whereas the drug did not affect the number of Ki67-positive cells in non-deprived rats. Similarly, mifepristone treatment tended to cause higher levels of DCX-positive cells in the dentate supra-pyramidal blade of MD rats compared to vehicle treated MD controls, although this did not reach significance (*p* = 0.08); mifepristone did not affect the number of DCX-positive cells in non-deprived rats. It should be noted that the effects were modest, possibly due to the age (PND 29) at which the effects of maternal deprivation were determined. More definite conclusions about the potential of mifepristone to reverse effects of maternal deprivation on cell proliferation and neurogenesis require extension of the current pilot experiment to analysis at an earlier time-point – when effects on neurogenesis are more clearly discernable in females, e.g., at PND 21, combined with mifepristone treatment at an earlier time-point, too. Nevertheless, the results are generally in line with earlier findings that brief treatment with the GR-antagonist mifepristone can quickly normalize effects of stress on neurogenesis (77).

## Concluding Remarks

Rodent studies over the past decades have shown that neurogenesis appears to be very sensitive to stress, particularly when stress occurs during the perinatal period. As has become evident from the current overview, these effects of perinatal stress are clearly age-dependent: the consequences seem to change in nature depending on the interval between early life adversity and the time of analysis of the effects on neurogenesis. Interestingly, the effects of perinatal stress on neurogenesis are also sex-dependent. Male rats show a brief period in adolescence during which neurogenesis, BDNF expression, and spatial learning are actually improved, possibly allowing the individual to temporarily compensate for the effects of early life adversity. Female rats do not show such a period of improved performance but rather show a very strong suppression of neurogenesis during the pre-pubertal period, which then subsides with age. The consequences of this period of suppressed neurogenesis in females, though, may be long-lasting. For instance, female rats exposed to 24 h of maternal deprivation at PND 3 exhibited a lower total number of mature granule cells in adulthood (71), potentially limiting the number of synaptic contacts that can be established in this region. Preliminary studies indicate that intervention at the pre-pubertal stage is possible, e.g., by blocking GRs for a limited number of days. Clearly, these studies on successful intervention strategies require more extensive follow-up, to precisely determine the effectiveness of various treatment regimes.

One can speculate about the implications of findings in animal models of perinatal stress for human brain development and the vulnerability to psychopathology. In general, the study of actual neurogenesis in the human brain is difficult and, although its existence has been convincingly demonstrated, it has largely relied on immunocytochemical approaches using proliferation markers in postmortem tissues. Although neurogenesis in adult and aged individuals is generally rare, much larger levels are present at earlier ages (85, 86). It is currently thought that antidepressant treatment may target the neurogenetic process and in fact requires the newborn cells for their antidepressant action to be exerted (87), although this is mostly based on studies in young animal models (60). The finding that especially female rats showed suppressed neurogenesis during a critical developmental stage in response to early life adversity is of interest, given the higher prevalence of many psychiatric illnesses in the female population. In humans, careful monitoring of genetically predisposed females with a history of early life adversity during their development, including possibilities for early intervention, may therefore be one approach to mitigate the development of psychopathology.

## Conflict of Interest Statement

The authors declare that the research was conducted in the absence of any commercial or financial relationships that could be construed as a potential conflict of interest.
